# Anti-NMDAR Encephalitis With Serial Negative MRI Findings: An Evaluation Using Autoimmune Psychosis Criteria

**DOI:** 10.1155/crnm/4561447

**Published:** 2025-06-02

**Authors:** Satoshi Saito, Go Taniguchi, Chihiro Nakata, Hideo Kato, Mao Otake, Masahiro Umeda, Yuichiro Fuji, Eiji Nakagawa

**Affiliations:** ^1^Department of Epileptology, National Center Hospital, National Center of Neurology and Psychiatry, 4-1-1 Ogawahigashi-cho, Kodaira, Tokyo 187-8551, Japan; ^2^Department of Neurology, Tokyo Women's Medical University School of Medicine, 8-1, Kawada-cho, Shinjuku-ku, Tokyo 162-8666, Japan

**Keywords:** altered consciousness, electroencephalography, levetiracetam, peri-ictal psychosis

## Abstract

Autoimmune psychosis criteria have been proposed for autoimmune encephalitis with prominent psychiatric symptoms as an alternative to biomarker-based diagnostic approaches such as the Graus criteria. We present a case of anti-N-methyl-D-aspartate receptor (NMDAR) encephalitis that was initially misdiagnosed as a psychiatric disorder due to serial negative MRI findings and subsequently re-evaluated correctly using autoimmune psychosis criteria. A 15-year-old male developed sudden-onset generalized convulsive seizures that increased progressively in frequency and fluctuating psychiatric symptoms that gradually worsened to include reduced reactivity, language deterioration, and catatonia. On admission, both brain MRI and cerebral spinal fluid (CSF) findings were unremarkable; however, autoimmune encephalitis was strongly suspected based on autoimmune psychosis criteria and subsequently confirmed by detection of oligoclonal bands (OCBs) and anti-NMDAR antibodies in the serum and CSF. Repeated steroid pulse therapy resulted in significant clinical improvement. The patient met multiple autoimmune psychosis criteria, including subacute onset of psychiatric symptoms, catatonia, disproportionate cognitive dysfunction, decreased level of consciousness, and the emergence of seizures. These features are not typically present in primary psychiatric disorders. Anti-NMDAR encephalitis can present with a variety of symptoms, complicating its differentiation from primary psychiatric conditions. The application of autoimmune psychosis criteria may serve as a valuable diagnostic aid, particularly when MRI findings are repeatedly negative.

## 1. Introduction

Timely diagnosis of autoimmune encephalitis is essential for preventing irreversible sequelae and reducing mortality risk [1]. The Graus diagnostic criteria emphasize a subacute clinical history along with acute biomarkers such as magnetic resonance imaging (MRI), cerebrospinal fluid (CSF) abnormalities, and electroencephalographic (EEG) findings [2]. However, a substantial proportion of patients with autoimmune encephalitis, including patients with anti-N-methyl-D-aspartate receptor (NMDAR) encephalitis, initially present with negative MRI findings [3]. In autoimmune encephalitis cases with negative MRI findings, autoantibody testing for definitive diagnosis may be delayed, potentially compromising clinical outcome. Moreover, few reports have analyzed the clinical course of autoimmune encephalitis with negative MRI findings, raising questions about whether reliance on conventional biomarkers (such as those included in the Graus criteria) is the most effective diagnostic approach.

A retrospective study of 65 anti-NMDAR encephalitis cases reported that the majority (59%) initially presented with psychiatric symptoms. These included visual or auditory hallucinations (40%), depression (23%), mania (8%), acute schizoaffective episodes (23%), and eating disorders or addiction (6%) [4]. When psychiatric symptoms predominate, treatment initiation is often delayed, leading to poorer prognosis [1, 4, 5]. Autoimmune psychosis criteria have been proposed for cases with prominent psychiatric symptoms [6]. However, these have not yet been fully validated for clinical efficacy.

In the present case, anti-NMDAR encephalitis was misdiagnosed as a psychiatric disorder due to serially negative MRI findings but subsequently diagnosed correctly based on autoimmune psychosis criteria and serology. We discuss the factors that may delay treatment and the potential for resolution using autoimmune psychosis criteria.

## 2. Case Presentation

**Figure 1:**
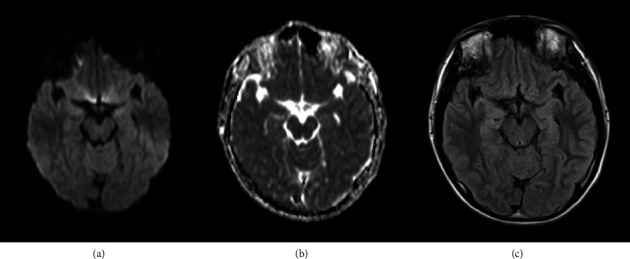
Normal axial magnetic resonance imaging (MRI) findings in the mesial temporal lobe on day 5 after symptom onset. (a) Axial diffusion-weighted imaging (DWI). (b) Axial apparent diffusion coefficient imaging. (c) Axial fluid-attenuated inversion recovery (FLAIR) imaging.

**Figure 2:**
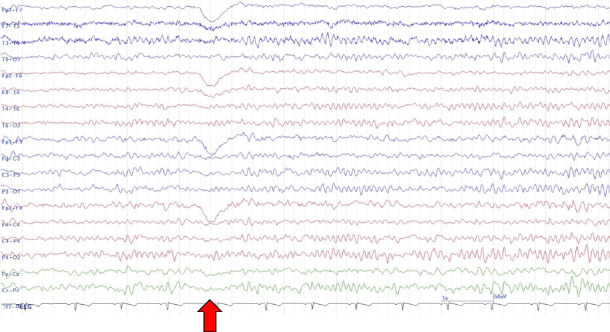
Electroencephalographic (EEG) findings on day 6 after symptom onset showing no epileptiform activity. The posterior dominant rhythm (8-9 Hz) was maintained, but organization of the background activity was mildly disordered. The red arrows indicate the initiation of eye closure. Alpha blocking was weak or absent during eye opening. The EEG conditions were as follows: Bipolar montage, high-pass filtering at 60 Hz, and time constant of 0.1 s.

The case patient was a 15-year-old male with mild intellectual disability and autism spectrum disorder but independence in activities of daily living. There was no history of perinatal abnormalities, no personal or family history of neurological or psychiatric diseases, and no prior relevant medication usage. Five days prior to hospital presentation, the patient began to exhibit episodes of impaired awareness characterized by staring at a point and unresponsiveness to external stimuli. At other times, the patient was oriented and able to communicate normally with family members. The following day, the patient experienced a generalized convulsion, followed by another seizure episode 3 days later, necessitating hospital admission. At the time of admission, levetiracetam (LEV) 1000 mg was initiated. Multimode brain MRI, including diffusion-weighted imaging (DWI), apparent diffusion coefficient mapping, and fluid-attenuated inversion recovery (FLAIR) sequences, revealed no abnormalities (Figure 1). On the sixth day postonset, the patient exhibited atypical violent behavior, including uttering abusive language and punching the wall, which resulted in hospital discharge. On the ninth day following onset (3 days after discharge), the patient attended an outpatient follow-up visit during which communication was limited to single words. Furthermore, the patient was unable to respond appropriately and exhibited purposeless behaviors such as repeated head hitting. An EEG examination revealed background activity (8-9 Hz) predominantly in the occipital area, weak alpha blocking during eye opening, and mildly disrupted EEG organization, but no epileptic abnormalities (Figure 2). Suspecting that the psychiatric symptoms were induced by LEV, the patient was switched to lacosamide (LCM) 300 mg.

**Figure 3:**
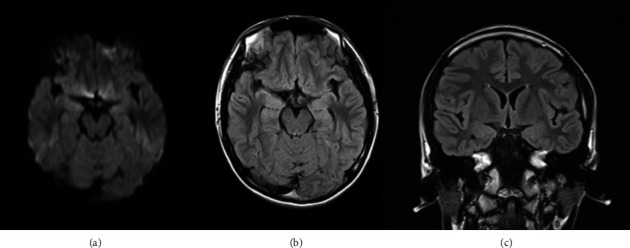
Normal magnetic resonance imaging (MRI) findings in the mesial temporal lobe on Day 24 after symptom onset. (a) Axial diffusion-weighted imaging (DWI). (b) Axial fluid-attenuated inversion recovery (FLAIR) imaging. (c) Coronal FLAIR imaging.

Despite antiepileptic treatment and negative MRI findings, the patient became completely nonverbal, began wandering aimlessly around the room, and occasionally froze in place with a blank expression. On Day 24 postonset, DWI and FLAIR sequences again revealed no detectable abnormalities (Figure 3), suggesting epileptic peri-ictal psychosis. Concurrently, the patient began to exhibit daily tonic seizures involving the right limbs and often lasting 2-3 min, after which consciousness gradually improved.

On Day 30 postonset, the patient was referred to our epilepsy clinic for the management of psychiatric symptoms and adjustment of antiseizure medication. At the time of examination, the patient was wheelchair-bound, mute, and maintained a fixed posture, showing minimal response to verbal stimuli. During examination, a seizure occurred, characterized by backward leaning, tonic stiffening of the right limbs, and upward rolling of the eyeballs. After approximately 1 min, the patient lost muscle tone and urinary continence. During the episode, SpO_2_ dropped to 90% and heart rate increased to 136 bpm.

**Figure 4:**
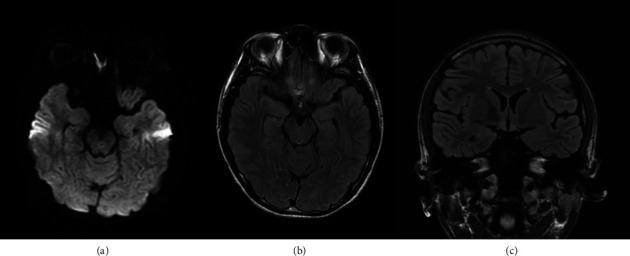
Normal magnetic resonance imaging (MRI) findings in the mesial temporal lobe on Day 30 after symptom onset. (a) Axial diffusion-weighted imaging (DWI). (b) Axial fluid-attenuated inversion recovery (FLAIR) imaging. (c) Coronal FLAIR imaging.

Blood tests revealed elevated serum creatine kinase levels (4760 U/L), consistent with convulsive seizures, but no metabolic abnormalities that could explain the seizure syndrome. Cerebrospinal fluid examination findings were also normal (cell count 1/μL, total protein 20 mg/dL, glucose 68 mg/dL, and IgG index 0.48) and further brain MRI findings, including DWI, T2-weighted, FLAIR, contrast-enhanced T1WI, and contrast-enhanced FLAIR sequences, still revealed no abnormalities (Figure 4).

Continuous sedation with midazolam was initiated together with antiseizure treatment using a mixture of LEV 1000 mg, LCM 200 mg, and perampanel 2 mg. However, tonic seizures of the right facial region and limbs continued. On Day 37 postonset, positive oligoclonal bands (OCBs) were identified, suggesting autoimmune encephalitis, and steroid pulse therapy (1000 mg/day for 5 days) was administered in three courses. During the course of steroid therapy, the level of consciousness improved, and seizure frequency gradually decreased. Subsequent anti-NMDAR antibody testing revealed positive results for both serum (10-fold increase) and CSF (20-fold increase), confirming anti-NMDAR encephalitis.

**Figure 5:**
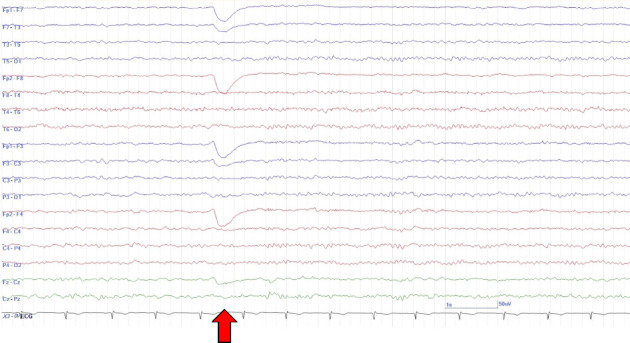
Electroencephalographic (EEG) findings on Day 80 after symptom onset showing no epileptiform activity. The posterior dominant rhythm (10-11 Hz) was maintained. The red arrows indicate the beginning of eye closure. Alpha blocking was strong during eye opening. The EEG conditions were as described in the legend for Figure 2.

On Day 80 postonset, the EEG showed normal background activity (10-11 Hz), reliable alpha blocking during eye opening, and no epileptic abnormalities (Figure 5). The patient was discharged home with independent ADL, and intellectual function eventually returned to baseline levels.

## 3. Discussion

Repeated negative MRI findings were a major factor contributing to delayed diagnosis in this case. While MRI is a crucial diagnostic tool for autoimmune encephalitis, a recent study of 37 autoimmune encephalitis patients found that nearly half (18 or 49%) exhibited no abnormal MRI findings during the disease course, including two cases of NMDAR encephalitis [7]. Thus, clinicians should not exclude the possibility of autoimmune encephalitis in cases with rapid onset psychiatric symptoms and convulsive seizures even when repeated MRI findings are unremarkable.

EEG is another important tool for diagnosing autoimmune encephalitis. Abnormal EEG findings are reported in 88.6%–98.4% of patients with anti-NMDAR encephalitis [8, 9], including epileptiform discharges, diffuse or focal slowing, polymorphic delta rhythms, diffuse beta activity, and extreme delta brush [9]. However, one study found normal occipital activity in 71% of initial EEGs from patients with anti-NMDAR encephalitis [10]. In our case, background EEG activity exhibited the posterior dominant rhythm within the normal frequency range, with mild disorganization and incomplete alpha blocking during eye opening. These findings were not strongly suggestive of autoimmune encephalitis. Given the sensitivity of EEG and the sporadic nature of abnormal activity [8, 9], repeated EEG is recommended as this may provide diagnostic clues for autoimmune encephalitis.

However, epilepsy-related conditions may also complicate the diagnosis of autoimmune encephalitis. The anticonvulsant LEV is known to cause psychotic symptoms in rare cases (0.6% in one study [11]), and seizures may be associated with peri-ictal psychosis and confusion [12]. In the current case, these conditions did complicate the diagnostic process and initially led to a psychiatric disorder diagnosis, potentially exacerbated by LEV. These conditions are generally not fatal and often improve with seizure control, withdrawal of suspected psychosis-inducing drugs, and (or) administration of psychotropic medications. Nonetheless, the exclusion of autoimmune encephalitis by serological testing should always be a priority.

The patient's initial psychiatric symptoms included intermittent affective disturbances, such as irritability. Over time, reactivity declined, while thought disorder became more pronounced and both speech and vocabulary deteriorated. Early negative psychiatric symptoms in anti-NMDAR encephalitis, such as reduced spontaneity, limited conversational fluency, and disorientation [13], may reflect impaired consciousness. Furthermore, in this case, catatonic features were also observed, including stupor, mutism, and posturing. Catatonia is not only specific to primary psychiatric disorders such as depression, mania, schizophrenia, and autism spectrum disorder but is also associated with organic disorders including autoimmune encephalitis, systemic lupus erythematosus, and thyroid dysfunction [14]. Anti-NMDAR encephalitis is the most commonly reported cause of autoimmune catatonia, occurring in up to 70% of affected patients [15]. Catatonia in anti-NMDAR encephalitis is thought to result from glutamatergic hypofunction [16]. In this case, the patient met multiple autoimmune psychosis criteria, including subacute onset of psychiatric symptoms, catatonia, disproportionate cognitive dysfunction, decreased level of consciousness, and the emergence of seizures, while none of these features are typically observed in primary psychiatric disorders [6].

The present case did not meet the Graus criteria but did fulfill the clinical symptom components of the autoimmune psychosis criteria. Therefore, testing for OCB and anti-NMDAR antibodies in serum and CSF should have been performed at presentation or soon thereafter despite negative brain MRI findings. Anti-NMDAR encephalitis can present with a wide range of symptoms; careful observation is essential to identify distinguishing features. Altered consciousness, worsening epileptic seizures, and catatonia are atypical of primary psychiatric disorders—particularly the emergence of seizures—and should prompt early consideration of autoimmune encephalitis. The application of autoimmune psychosis criteria may thus serve as an important diagnostic aid.

## Data Availability

The data that support the findings of this study are available from the corresponding author upon reasonable request.
